# Supplementary material for: Target enrichment of ultraconserved elements from arthropods provides a genomic perspective on relationships among Hymenoptera
Source: Mol Ecol Resour. 2014 Sep 29;15(3):489–501. doi: 10.1111/1755-0998.12328 (PMC4407909; doi:10.1111/1755-0998.12328)
Supplement: Supplementary file 1 — Table S1Family, species, collection identifier, collection year, collection country, collection method, voucher identifier, voucher depository, total amount of extract DNA, amount of DNA input to library preparation, post-enrichment method, and MiSeq run of all samples used for target enrichment. Table S2 Species, genome assembly, genome assembly source, reference, and number of UCE loci in assembly for all genome-enabled taxa. Table S3 Quantitative PCR primers used for assessment (relative quantification) of enrichment success and enrichment differences of Cot-1 sources. Table S4 Crossing point (Cp) values for quantitative PCR showing the fold enrichment differences between unenriched controls, enrichments using chicken Cot-1 as a blocking agent, enrichments using hymenoptera Cot-1 as a blocking agent, and Δ Cot-1 or the fold-enrichment difference between chicken and hymenoptera Cot-1. Table S5 Summary values describing the number of reads collected during sequencing of each enriched library. Table S6 Summary values describing the number of contigs assembled by ABySS from adapter- and quality-trimmed reads (“All” contigs), their average coverage, the mean length of All contigs, the count of unique reads aligned to All contigs, the number of UCE contigs identified from the pool of All contigs, the mean length of UCE contigs, the average UCE contig sequencing coverage, and the percentage of unique reads that aligned to UCE contigs (this is a percentage of the percentage of unique reads aligning to All contigs). Table S7 Summary values describing attributes of the UCE contigs assembled by Trinity. Table S8 Summary values describing attributes of the UCE contigs assembled by ABySS. Table S9 Model structure, AIC, number of parameters, AICc, and Akaike weight (wi) for general linear models of parameters affecting the mean number of UCE contigs captured. Table S10 Model structure, AIC, number of parameters, AICc, and Akaike weight (wi) for general linear models of paramet [file men0015-0489-sd1.docx]

**Supplemental Table 1**: Family, species, collection identifier, collection year, collection country, collection method, voucher identifier, voucher depository, total amount of extract DNA, amount of DNA input to library preparation, post-enrichment method, and MiSeq run of all samples used for target enrichment.

| **Family** | **Species** | **CollnID** | **CollnYr** | **Country** | **CollnMethod** | **VoucherID** | **Deposited** | **DNA (ng)** | **Lib DNA (ng)** | **Post enrichment method** | **MiSeq Run** |
| --- | --- | --- | --- | --- | --- | --- | --- | --- | --- | --- | --- |
| Andrenidae | *Andrena (Callandrena) asteris* | USGS_DRO_137659 | 2009 | USA | hand net | USNMENT00921242 | USNM | 6480 | 486 | with bead | R1 |
| Andrenidae | *Andrena (Melandrena) sp* | None | 2012 | USA | Malaise trap | USNMENT00921243 | USNM | 693 | 69 | with bead | R1 |
| Apidae | *Bombus pensylvanicus* | USGS_DRO_13724 | 2009 | USA | soapy pan trap | USNMENT00921244 | USNM | 560 | 470 | with bead | R1 |
| Bradynobaenidae | *Chyphotes mellipes* | PSW14654 | 2002 | USA | unknown | CASENT0106101 | CASC | 678 | 498 | with bead | R1 |
| Evaniidae | *Evaniella semaeoda* | D. Smith 5 | 2012 | USA | Malaise trap | USNMENT00921245 | USNM | 2340 | 503 | with bead | R1 |
| Formicidae | *Aphaenogaster albisetosa* | MGB1978 | 2011 | USA | hand collection | USNMENT00921246 | USNM | 784 | 500 | NaOH | R2 |
| Formicidae | *Aphaenogaster megommata* | PSW16689 | 2012 | Mexico | hand collection | USNMENT00921247 | USNM | 319 | 266 | NaOH | R2 |
| Formicidae | *Aphaenogaster tennesseensis* | MGB2040 | 2013 | USA | hand collection | USNMENT00921248 | USNM | 779 | 500 | NaOH | R2 |
| Formicidae | *Aphaenogaster texana* | MGB1983 | 2011 | USA | litter sifting | USNMENT00921249 | USNM | 736 | 500 | NaOH | R2 |
| Formicidae | *Messor piceus* | TRP-2012a-NPHC(B) | 2012 | S. Africa | hand collection | USNMENT00921250 | USNM | 932 | 500 | NaOH | R2 |
| Formicidae | *Pogonomyrmex occidentalis* | MGB2005 | 2012 | USA | hand collection | USNMENT00921251 | USNM | 1021 | 500 | NaOH | R2 |
| Formicidae | *Sericomyrmex harekulli* | AJ111125-05 | 2011 | Guyana | bait | USNMENT00921252 | USNM | 843 | 497 | with bead | R1 |
| Formicidae | *Stenamma diecki* | MGB2046 | 2013 | USA | hand collection | USNMENT00921253 | USNM | 284 | 237 | NaOH | R2 |
| Formicidae | *Stenamma expolitum* | MGB1880 | 2011 | Nicaragua | hand collection | USNMENT00921254 | USNM | 400 | 333 | NaOH | R2 |
| Formicidae | *Stenamma felixi* | JTL7524 | 2011 | Nicaragua | hand collection | USNMENT00921255 | USNM | 533 | 444 | NaOH | R2 |
| Formicidae | *Stenamma impar* | MGB2036 | 2013 | USA | hand collection | USNMENT00921256 | USNM | 262 | 218 | NaOH | R2 |
| Formicidae | *Stenamma megamanni* | Wm-D-05-1-01 | 2011 | Nicaragua | litter sifting | USNMENT00921257 | USNM | 315 | 252 | with bead | R1 |
| Formicidae | *Stenamma megamanni* | MGB1764 | 2011 | Nicaragua | hand collection | USNMENT00921258 | USNM | 326 | 272 | NaOH | R2 |
| Formicidae | *Stenamma muralla* | JTL7007 | 2010 | Honduras | hand collection | USNMENT00921259 | USNM | 181 | 151 | NaOH | R2 |
| Pergidae | *Acordulecera pellucida* | D. Smith 3 | 2012 | USA | Malaise trap | USNMENT00921260 | USNM | 1060 | 424 | with bead | R1 |
| Pompilidae | *Aporus niger* | ANTC4004 | 2002 | USA | unknown | CASENT0106104 | CASC | 2120 | 498 | with bead | R1 |
| Pteromalidae | *Nasonia vitripennis*** | None | 2013 | USA | purchased | None | None | 3923 | 500 | with bead | R1 |
| Sapygidae | *Sapyga pumila* | ANTC4005 | 2004 | USA | unknown | CASENT0106105 | CASC | 2270 | 499 | with bead | R1 |
| Scoliidae | *Scolia verticalis* | ANTC4007 | 2004 | Australia | unknown | CASENT0106107 | CASC | 5550 | 500 | with bead | R1 |
| Sphecidae | *Chalybion californicum* | PSW15440 | 2005 | USA | unknown | CASENT0106103 | CASC | 3730 | 506 | with bead | R1 |
| Tenthredinidae | *Nematus tibialis* | D. Smith 2 | 2012 | USA | Malaise trap | USNMENT00921261 | USNM | 2950 | 502 | with bead | R1 |
| Tenthredinidae | *Taxonus pallidicornis* | D. Smith 1 | 2012 | USA | Malaise trap | USNMENT00921262 | USNM | 6120 | 490 | with bead | R1 |
| Trigonalidae | *Orthogonalys pulchella* | D. Smith 4 | 2012 | USA | Malaise trap | USNMENT00921263 | USNM | 3390 | 509 | with bead | R1 |
| Vespidae | *Metapolybia cingulata* | ANTC4006 | 2007 | Peru | unknown | CASENT0106106 | USNM | 749 | 502 | with bead | R1 |
| Vespidae | *Mischocyttarus flavitarsis* | PSW15442 | 2005 | USA | unknown | CASENT0106102 | USNM | 6480 | 486 | with bead | R1 |

**Supplemental Table 2**: Species, genome assembly, genome assembly source, reference, and number of UCE loci in assembly for all genome-enabled taxa.

| **Species** | **Name of assembly** | **Assembly source** | **Reference** | **Unique UCE loci in assembly** |
| --- | --- | --- | --- | --- |
| *Acromyrmex echinatior* | Aech_3.9 | Genbank | 10.1101/gr.121392.111 | 774 |
| *Apis mellifera* | Amel_4.5 | Genbank | 10.1186/1471-2164-15-86 | 803 |
| *Atta cephalotes* | Attacep1.0 | Genbank | 10.1371/journal.pgen.1002007 | 748 |
| *Camponotus floridanus* | CamFlo_1.0 | Genbank | 10.1126/science.1192428 | 767 |
| *Cerapachys biroi* | CerBir1.0 | Genbank | 10.1016/j.cub.2014.01.018 | 768 |
| *Ceratosolen solmsi* | CerSol_1.0 | Genbank | 10.1186/gb-2013-14-12-r141 | 897 |
| *Harpegnathos saltator* | HarSal_1.0 | Genbank | 10.1126/science.1192428 | 763 |
| *Lasioglossum albipes* | ASM34657v1 | Genbank | 10.1186/gb-2013-14-12-r142 | 779 |
| *Linepithema humile* | Lhum_1.0 | hymenopteragenome.org | 10.1073/pnas.1008617108 | 762 |
| *Nasonia giraulti* | Ngir_1.0 | Genbank | 10.1126/science.1178028 | 1191 |
| *Nasonia longicornis* | Nlon_1.0 | Genbank | 10.1126/science.1178028 | 1192 |
| *Nasonia vitripennis* | Nvit_2.0 | Genbank | 10.1126/science.1178028 | 1214 |
| *Pogonomyrmex barbatus* | Pbar_UMD_V03 | Genbank | 10.1073/pnas.1007901108 | 666 |
| *Solenopsis invicta* | Si_gnG | Genbank | 10.1073/pnas.1009690108 | 768 |

**Supplemental Table 3**: Quantitative PCR primers used for assessment (relative quantification) of enrichment success and enrichment differences of Cot-1 sources.

| **UCE locus** | **UCE locus genomic position** | **UCE locus size** | **Upper primer (5' - 3')** | **Tm upper primer** | **Lower primer (5' - 3')** | **Tm lower primer** | **Estimated product size** |
| --- | --- | --- | --- | --- | --- | --- | --- |
| uce-82 | chr1:2966279-2966458 | 114 | GCCGACCCCCTGCTGAAGAG | 59.1 | AGACTTACGGCGTCTGCCACG | 59.2 | 77 |
| uce-202 | chr2:4442225-4442404 | 170 | GCCATGCGTGTTCGCTCTTGC | 59.9 | TGCATCGGCCCTTGACAGCG | 60 | 162 |
| uce-591 | chr2:34873617-34873796 | 136 | GGGCATCTACACATTTGAGTCCGCC | 59.9 | ACGAAGTCGAGCCAATTCCATGC | 58 | 102 |
| uce-1101 | chr4:34336396-34336576 | 127 | CGTAGCCATAACGATCGGTCGCC | 59.8 | ACACACCACTGTCGGACAAACTGC | 59.8 | 87 |
| uce-1160 | chr4:5470676-5470856 | 125 | AGGCTTTGGGTGGGCGTTCG | 59.9 | TCACAGCACACACTGGGCCG | 59.6 | 121 |
| uce-1196 | chr4:4001320-4001500 | 137 | GATTAGGGTTGGGGCCTAGGACAGG | 59.8 | GGGGGACAGTACGTGGCTCG | 58.9 | 75 |
| uce-1481 | ChrUn.Scaffold477:51625-51805 | 119 | TCTTCTGCATGGCGTGGTTGG | 57.7 | ACAAGTGCGCTTGCAATTTGTTGGG | 57.7 | 75 |

**Supplemental Table 4**: Crossing point (C_p_) values for quantitative PCR showing the fold enrichment differences between unenriched controls, enrichments using chicken Cot-1 as a blocking agent, enrichments using hymenoptera Cot-1 as a blocking agent, and Δ Cot-1 or the fold-enrichment difference between chicken and hymenoptera Cot-1.

|  | **Unenriched controls** | |  | **Used chicken Cot-1** | | | |  | **Used hymenoptera Cot-1** | | | |  | **Δ Cot-1** |
| --- | --- | --- | --- | --- | --- | --- | --- | --- | --- | --- | --- | --- | --- | --- |
| **Pool 1** | **Name** | **Cp** |  | **Name** | **Cp** | **Delta Cp** | **Enrichment** |  | **Name** | **Cp** | **Delta Cp** | **Enrichment** |  |  |
|  | c1-1162 | 21.23 |  | p1c-1162 | 13.9 | 7.3 | 158.7 |  | p1h-1162 | 14.4 | 6.8 | 51.0 |  | 107.6 |
|  | c1-132 | 27.27 |  | p1c-132 | 17.9 | 9.3 | 643.6 |  | p1h-132 | 18.2 | 9.1 | 184.6 |  | 459.0 |
|  | c1-2055 | 21.82 |  | p1c-2055 | 13.7 | 8.1 | 280.1 |  | p1h-2055 | 14.3 | 7.6 | 78.6 |  | 201.5 |
|  | c1-2118 | 21.27 |  | p1c-2118 | 16.5 | 4.8 | 27.3 |  | p1h-2118 | 16.9 | 4.4 | 12.6 |  | 14.6 |
|  | c1-2173 | 21.47 |  | p1c-2173 | 13.9 | 7.5 | 184.8 |  | p1h-2173 | 14.8 | 6.7 | 46.5 |  | 138.3 |
|  | c1-2704 | 22.10 |  | p1c-2704 | 14.3 | 7.8 | 227.5 |  | p1h-2704 | 15.0 | 7.1 | 59.6 |  | 167.9 |
|  | c1-539 | 27.78 |  | p1c-539 | 18.6 | 9.2 | 580.0 |  | p1h-539 | 19.6 | 8.2 | 110.5 |  | 469.5 |
|  | c1-neg |  |  | p1c-neg |  |  |  |  | p1h-neg |  |  |  |  |  |
|  |  |  |  |  |  |  |  |  |  |  |  |  |  |  |
| **Pool 2** | c2-1162 | 22.41 |  | p2c-1162 | 13.6 | 8.8 | 455.1 |  | p2h-1162 | 14.3 | 8.1 | 108.0 |  | 347.1 |
|  | c2-1162-r2 | 22.26 |  | p2c-1162-r2 | 13.4 | 8.8 | 458.3 |  | p2h-1162-r2 | 14.0 | 8.2 | 114.4 |  | 343.8 |
|  | c2-132 | 27.56 |  | p2c-132 | 17.5 | 10.1 | 1067.5 |  | p2h-132 | 18.7 | 8.9 | 168.4 |  | 899.1 |
|  | c2-132-r2 | 27.29 |  | p2c-132-r2 | 17.3 | 10.0 | 1031.1 |  | p2h-132-r2 | 17.5 | 9.8 | 284.5 |  | 746.6 |
|  | c2-2055 | 23.23 |  | p2c-2055 | 13.5 | 9.7 | 855.1 |  | p2h-2055 | 14.0 | 9.2 | 200.2 |  | 655.0 |
|  | c2-2055-r2 | 22.79 |  | p2c-2055-r2 | 13.4 | 9.4 | 661.7 |  | p2h-2055-r2 | 14.0 | 8.8 | 163.6 |  | 498.1 |
|  | c2-2118 | 23.51 |  | p2c-2118 | 17.1 | 6.4 | 86.8 |  | p2h-2118 | 17.8 | 5.7 | 27.2 |  | 59.6 |
|  | c2-2118-r2 | 23.22 |  | p2c-2118-r2 | 17.0 | 6.3 | 76.6 |  | p2h-2118-r2 | 17.6 | 5.7 | 26.0 |  | 50.6 |
|  | c2-2173 | 23.76 |  | p2c-2173 | 14.6 | 9.1 | 564.2 |  | p2h-2173 | 15.3 | 8.5 | 132.1 |  | 432.0 |
|  | c2-2173-r2 | 23.56 |  | p2c-2173-r2 | 14.4 | 9.1 | 560.3 |  | p2h-2173-r2 | 15.0 | 8.5 | 136.0 |  | 424.3 |
|  | c2-2704 | 23.52 |  | p2c-2704 | 14.5 | 9.0 | 515.6 |  | p2h-2704 | 15.3 | 8.2 | 114.4 |  | 401.2 |
|  | c2-2704-r2 | 23.55 |  | p2c-2704-r2 | 14.5 | 9.0 | 519.1 |  | p2h-2704-r2 | 14.9 | 8.6 | 144.9 |  | 374.2 |
|  | c2-539 | 31.04 |  | p2c-539 | 20.0 | 11.0 | 2091.0 |  | p2h-539 | 21.0 | 10.1 | 336.3 |  | 1754.7 |
|  | c2-539-r2 | 31.07 |  | p2c-539-r2 | 19.6 | 11.5 | 2916.5 |  | p2h-539-r2 | 20.1 | 10.9 | 549.0 |  | 2367.4 |
|  | c2-neg |  |  | p2c-neg |  |  |  |  | p2h-neg |  |  |  |  |  |
|  | c2-neg-r2 |  |  | p2c-neg-r2 |  |  |  |  | p2h-neg-r2 |  |  |  |  |  |
|  |  |  |  |  |  |  |  |  |  |  |  |  |  |  |
| **Pool 3** | c3-1162 | 22.90 |  | p3c-1162 | 14.0 | 8.9 | 474.4 |  | p3h-1162 | 14.3 | 8.6 | 145.8 |  | 328.7 |
|  | c3-132 | 28.58 |  | p3c-132 | 17.7 | 10.9 | 1910.9 |  | p3h-132 | 18.0 | 10.6 | 448.7 |  | 1462.2 |
|  | c3-2055 | 23.29 |  | p3c-2055 | 13.7 | 9.6 | 792.4 |  | p3h-2055 | 13.8 | 9.5 | 240.7 |  | 551.6 |
|  | c3-2118 | 23.18 |  | p3c-2118 | 16.0 | 7.2 | 149.1 |  | p3h-2118 | 16.6 | 6.6 | 43.9 |  | 105.2 |
|  | c3-2173 | 23.70 |  | p3c-2173 | 14.1 | 9.6 | 797.9 |  | p3h-2173 | 14.3 | 9.5 | 232.5 |  | 565.3 |
|  | c3-2704 | 23.14 |  | p3c-2704 | 13.8 | 9.3 | 643.6 |  | p3h-2704 | 14.1 | 9.1 | 185.7 |  | 457.9 |
|  | c3-539 | 30.72 |  | p3c-539 | 19.7 | 11.0 | 2105.6 |  | p3h-539 | 19.5 | 11.2 | 641.5 |  | 1464.1 |
|  | c3-neg |  |  | p3c-neg |  |  |  |  | p3h-neg |  |  |  |  |  |
|  |  |  |  |  |  |  |  |  |  |  |  |  |  |  |
|  |  |  |  |  |  | **Avg.** | 744.1 |  |  |  | **Avg.** | 178.1 | **Avg.** | 566.0 |
|  |  |  |  |  |  | **95 CI** | 259.9 |  |  |  | **95 CI** | 57.1 | **95 CI** | 207.0 |

**Supplemental Table 5**: Summary values describing the number of reads collected during sequencing of each enriched library.

| **Taxon** | **Trimmed reads** | **Total BP** | **Mean length** | **95 % CI** | **Min lengths** | **Max length** | **Median length** |
| --- | --- | --- | --- | --- | --- | --- | --- |
| *Acordulecera pellucida* | 408,901 | 86,125,439 | 210.6 | 0.1 | 40 | 251 | 250 |
| *Andrena (Callandrena) asteris* | 83,975 | 15,540,798 | 185.1 | 0.2 | 40 | 251 | 199 |
| *Andrena (Melandrena) sp* | 410,453 | 84,592,764 | 206.1 | 0.1 | 40 | 251 | 232 |
| *Aphaenogaster albisetosa* | 2,217,687 | 426,328,391 | 192.2 | 0.0 | 40 | 251 | 207 |
| *Aphaenogaster megommata* | 2,047,669 | 386,980,038 | 189.0 | 0.0 | 40 | 251 | 198 |
| *Aphaenogaster tennesseensis* | 1,625,068 | 281,397,272 | 173.2 | 0.0 | 40 | 251 | 173 |
| *Aphaenogaster texana* | 1,059,887 | 182,422,784 | 172.1 | 0.1 | 40 | 251 | 171 |
| *Aporus niger* | 398,607 | 74,624,652 | 187.2 | 0.1 | 40 | 251 | 195 |
| *Bombus pensylvanicus* | 301,910 | 63,481,685 | 210.3 | 0.1 | 40 | 251 | 250 |
| *Chalybion californicus* | 654,184 | 117,929,426 | 180.3 | 0.1 | 40 | 251 | 183 |
| *Chyphotes mellipes* | 1,664,263 | 322,103,690 | 193.5 | 0.0 | 40 | 251 | 208 |
| *Evaniella semaeoda* | 414,086 | 78,104,105 | 188.6 | 0.1 | 40 | 251 | 202 |
| *Messor piceus* | 1,710,354 | 331,117,936 | 193.6 | 0.0 | 40 | 251 | 209 |
| *Metapolybia cingulata* | 719,460 | 142,797,714 | 198.5 | 0.1 | 40 | 251 | 220 |
| *Mischocyttarus flavitarsis* | 307,969 | 61,394,499 | 199.4 | 0.1 | 40 | 251 | 223 |
| *Nasonia vitripennis* | 528,367 | 99,597,773 | 188.5 | 0.1 | 40 | 251 | 199 |
| *Nematus tibialis* | 703,569 | 135,792,550 | 193.0 | 0.1 | 40 | 251 | 213 |
| *Orthogonalys pulchella* | 1,822,967 | 354,456,435 | 194.4 | 0.0 | 40 | 251 | 214 |
| *Pogonomyrmex occidentalis* | 2,129,915 | 406,383,752 | 190.8 | 0.0 | 40 | 251 | 203 |
| *Sapyga pumila* | 1,732,085 | 311,775,579 | 180.0 | 0.0 | 40 | 251 | 180 |
| *Scolia verticalis* | 907,356 | 178,554,253 | 196.8 | 0.1 | 40 | 251 | 221 |
| *Sericomyrmex harekulli* | 327,399 | 64,865,315 | 198.1 | 0.1 | 40 | 251 | 214 |
| *Stenamma diecki* | 1,579,469 | 314,662,462 | 199.2 | 0.0 | 40 | 251 | 218 |
| *Stenamma expolitum* | 1,847,383 | 362,261,429 | 196.1 | 0.0 | 40 | 251 | 211 |
| *Stenamma felixi* | 2,001,433 | 356,384,998 | 178.1 | 0.0 | 40 | 251 | 179 |
| *Stenamma impar* | 1,541,096 | 293,383,544 | 190.4 | 0.0 | 40 | 251 | 199 |
| *Stenamma megamanni* | 2,179,975 | 395,476,057 | 181.4 | 0.0 | 40 | 251 | 181 |
| *Stenamma megamanni2* | 801,435 | 169,627,489 | 211.7 | 0.1 | 40 | 251 | 250 |
| *Stenamma muralla* | 1,237,264 | 238,365,794 | 192.7 | 0.1 | 40 | 251 | 203 |
| *Taxonus pallidicornis* | 577,999 | 119,464,673 | 206.7 | 0.1 | 40 | 251 | 250 |

**Supplemental Table 6**: Summary values describing the number of contigs assembled by ABySS from adapter- and quality-trimmed reads (“All” contigs), their average coverage, the mean length of All contigs, the count of unique reads aligned to All contigs, the number of UCE contigs identified from the pool of All contigs, the mean length of UCE contigs, the average UCE contig sequencing coverage, and the percentage of unique reads that aligned to UCE contigs (this is a percentage of the percentage of unique reads aligning to All contigs).

| **Taxon** | **All contigs** | **All contigs  coverage** | **All contigs  coverage 95 CI** | **All contigs  mean length** | **All contigs  mean length 95 CI** | **All contigs  unique reads aligned** | **UCE contigs** | **UCE contigs  mean length** | **UCE contigs  coverage** | **UCE contigs  unique reads aligned** |
| --- | --- | --- | --- | --- | --- | --- | --- | --- | --- | --- |
| *Acordulecera pellucida* | 62,419 | 3.4 | 0.1 | 197.4 | 0.8 | 85.6% | 319 | 705.0 | 30.3 | 12.1% |
| *Andrena (Callandrena) asteris* | 9,027 | 4.6 | 0.3 | 208.6 | 2.4 | 79.3% | 714 | 437.1 | 11.5 | 36.9% |
| *Andrena (Melandrena) sp* | 69,660 | 3.3 | 0.2 | 194.1 | 0.7 | 88.2% | 704 | 636.9 | 19.9 | 17.4% |
| *Aphaenogaster albisetosa* | 275,539 | 4.1 | 0.0 | 218.5 | 0.4 | 87.4% | 302 | 654.7 | 43.9 | 2.8% |
| *Aphaenogaster megommata* | 230,940 | 4.1 | 0.1 | 201.2 | 0.4 | 81.0% | 323 | 725.1 | 46.8 | 4.3% |
| *Aphaenogaster tennesseensis* | 184,108 | 4.2 | 0.1 | 186.7 | 0.4 | 82.4% | 412 | 678.6 | 44.9 | 7.2% |
| *Aphaenogaster texana* | 126,796 | 4.0 | 0.1 | 179.4 | 0.4 | 82.6% | 348 | 522.8 | 30.3 | 5.1% |
| *Aporus niger* | 37,593 | 5.0 | 1.0 | 193.1 | 1.0 | 84.1% | 725 | 559.6 | 17.1 | 18.5% |
| *Bombus pensylvanicus* | 55,323 | 3.0 | 0.1 | 197.4 | 0.8 | 88.9% | 703 | 632.0 | 23.9 | 23.7% |
| *Chalybion californicus* | 91,078 | 3.5 | 0.2 | 196.6 | 0.6 | 80.1% | 660 | 614.1 | 34.1 | 18.1% |
| *Chyphotes mellipes* | 191,326 | 4.4 | 0.3 | 222.8 | 0.6 | 89.5% | 472 | 808.1 | 59.7 | 9.2% |
| *Evaniella semaeoda* | 43,255 | 4.4 | 0.2 | 193.8 | 1.1 | 85.9% | 515 | 702.8 | 30.9 | 22.1% |
| *Messor piceus* | 190,453 | 4.5 | 0.2 | 194.0 | 0.4 | 82.8% | 423 | 693.8 | 43.7 | 6.5% |
| *Metapolybia cingulata* | 131,497 | 3.1 | 0.0 | 197.7 | 0.5 | 88.3% | 562 | 618.4 | 38.2 | 13.2% |
| *Mischocyttarus flavitarsis* | 37,614 | 4.4 | 0.3 | 194.0 | 1.0 | 88.1% | 616 | 519.3 | 33.0 | 24.5% |
| *Nasonia vitripennis* | 69,994 | 3.8 | 0.1 | 180.0 | 0.6 | 87.0% | 756 | 463.1 | 45.5 | 22.8% |
| *Nematus tibialis* | 101,439 | 3.4 | 0.1 | 197.3 | 0.7 | 85.4% | 324 | 683.6 | 47.1 | 10.6% |
| *Orthogonalys pulchella* | 174,934 | 3.9 | 0.1 | 251.1 | 0.8 | 83.8% | 266 | 807.8 | 74.1 | 4.6% |
| *Pogonomyrmex occidentalis* | 266,414 | 3.9 | 0.0 | 220.1 | 0.4 | 86.2% | 293 | 658.2 | 56.6 | 3.1% |
| *Sapyga pumila* | 215,619 | 3.7 | 0.1 | 210.1 | 0.5 | 84.4% | 349 | 596.3 | 71.1 | 5.7% |
| *Scolia verticalis* | 105,754 | 3.9 | 0.1 | 208.1 | 0.7 | 84.4% | 516 | 794.1 | 49.2 | 16.2% |
| *Sericomyrmex harekulli* | 61,272 | 3.1 | 0.0 | 185.4 | 0.8 | 89.4% | 663 | 606.1 | 24.6 | 22.1% |
| *Stenamma diecki* | 194,733 | 4.0 | 0.1 | 207.0 | 0.5 | 85.5% | 523 | 820.2 | 46.6 | 10.0% |
| *Stenamma expolitum* | 236,274 | 3.9 | 0.0 | 214.7 | 0.5 | 86.9% | 376 | 785.6 | 50.3 | 5.4% |
| *Stenamma felixi* | 277,927 | 3.5 | 0.0 | 203.8 | 0.4 | 85.1% | 263 | 603.4 | 35.6 | 2.2% |
| *Stenamma impar* | 180,786 | 4.3 | 0.1 | 197.9 | 0.5 | 86.6% | 524 | 727.8 | 45.4 | 9.6% |
| *Stenamma megamanni* | 140,321 | 3.0 | 0.0 | 209.4 | 0.6 | 87.9% | 544 | 803.3 | 35.1 | 12.9% |
| *Stenamma megamanni2* | 285,366 | 3.5 | 0.0 | 213.0 | 0.4 | 86.3% | 314 | 765.0 | 45.6 | 3.8% |
| *Stenamma muralla* | 188,981 | 3.2 | 0.0 | 205.8 | 0.4 | 85.1% | 392 | 713.3 | 46.6 | 7.5% |
| *Taxonus pallidicornis* | 79,453 | 3.5 | 0.1 | 202.9 | 0.8 | 82.5% | 416 | 737.2 | 40.1 | 15.7% |

**Supplemental Table 7**: Summary values describing attributes of the UCE contigs assembled by Trinity.

| **Taxon** | **UCE contigs** | **UCE contigs total BP** | **UCE contigs mean length** | **UCE contigs mean length 95 CI** | **UCE contigs min length** | **UCE contigs max length** | **UCE contigs median length** | **UCE contigs > 1kb** | **UCE contigs  coverage** | **UCE contigs  unique reads aligned** |
| --- | --- | --- | --- | --- | --- | --- | --- | --- | --- | --- |
| *Acordulecera pellucida* | 341 | 349,519 | 1,025.0 | 19.7 | 206 | 2,504 | 1,054.0 | 198 | 26.3 | 18.4% |
| *Andrena (Callandrena) asteris* | 740 | 425,271 | 574.7 | 7.0 | 202 | 1,447 | 558.0 | 16 | 9.8 | 44.4% |
| *Andrena (Melandrena) sp* | 774 | 663,345 | 857.0 | 9.9 | 208 | 2,253 | 861.5 | 234 | 18.2 | 25.9% |
| *Aphaenogaster albisetosa* | 764 | 862,282 | 1,128.6 | 20.8 | 223 | 11,435 | 1,124.5 | 461 | 88.3 | 26.0% |
| *Aphaenogaster megommata* | 751 | 889,250 | 1,184.1 | 16.2 | 230 | 2,776 | 1,170.0 | 484 | 79.2 | 28.8% |
| *Aphaenogaster tennesseensis* | 751 | 793,300 | 1,056.3 | 14.8 | 210 | 2,493 | 1,051.0 | 412 | 62.4 | 30.5% |
| *Aphaenogaster texana* | 750 | 693,422 | 924.6 | 12.8 | 207 | 2,645 | 906.0 | 281 | 51.5 | 33.2% |
| *Aporus niger* | 740 | 528,399 | 714.1 | 9.1 | 205 | 1,981 | 717.0 | 71 | 14.5 | 17.7% |
| *Bombus pensylvanicus* | 780 | 670,544 | 859.7 | 9.2 | 206 | 2,036 | 879.0 | 228 | 21.4 | 35.0% |
| *Chalybion californicus* | 778 | 631,930 | 812.2 | 10.4 | 205 | 1,968 | 809.0 | 201 | 33.2 | 29.6% |
| *Chyphotes mellipes* | 774 | 916,382 | 1,184.0 | 13.0 | 294 | 3,189 | 1,185.0 | 558 | 66.2 | 26.6% |
| *Evaniella semaeoda* | 638 | 619,918 | 971.7 | 11.6 | 220 | 2,229 | 978.5 | 301 | 31.1 | 39.1% |
| *Messor piceus* | 730 | 811,543 | 1,111.7 | 15.7 | 210 | 3,730 | 1,119.5 | 441 | 58.9 | 26.2% |
| *Metapolybia cingulata* | 685 | 563,953 | 823.3 | 12.8 | 207 | 2,103 | 801.0 | 211 | 40.1 | 24.7% |
| *Mischocyttarus flavitarsis* | 634 | 450,896 | 711.2 | 11.6 | 203 | 2,687 | 676.5 | 110 | 30.0 | 32.4% |
| *Nasonia vitripennis* | 1,166 | 899,101 | 771.1 | 7.7 | 202 | 1,672 | 763.0 | 237 | 46.9 | 57.1% |
| *Nematus tibialis* | 453 | 475,444 | 1,049.5 | 17.9 | 209 | 3,894 | 1,070.0 | 265 | 47.9 | 26.4% |
| *Orthogonalys pulchella* | 706 | 962,959 | 1,364.0 | 16.5 | 205 | 2,998 | 1,352.0 | 569 | 109.0 | 35.0% |
| *Pogonomyrmex occidentalis* | 741 | 846,554 | 1,142.4 | 16.2 | 231 | 3,190 | 1,124.0 | 457 | 97.5 | 26.8% |
| *Sapyga pumila* | 720 | 753,734 | 1,046.9 | 13.4 | 224 | 2,743 | 1,078.0 | 428 | 86.4 | 28.6% |
| *Scolia verticalis* | 760 | 813,497 | 1,070.4 | 12.2 | 286 | 2,877 | 1,078.0 | 461 | 56.6 | 36.0% |
| *Sericomyrmex harekulli* | 744 | 606,204 | 814.8 | 9.7 | 205 | 2,099 | 830.5 | 177 | 22.3 | 33.5% |
| *Stenamma diecki* | 751 | 857,659 | 1,142.0 | 15.0 | 209 | 3,188 | 1,167.0 | 488 | 53.5 | 23.7% |
| *Stenamma expolitum* | 749 | 907,836 | 1,212.1 | 15.7 | 205 | 2,690 | 1,216.0 | 520 | 69.3 | 25.7% |
| *Stenamma felixi* | 762 | 816,726 | 1,071.8 | 14.2 | 209 | 3,469 | 1,056.0 | 433 | 75.3 | 25.1% |
| *Stenamma impar* | 741 | 782,478 | 1,056.0 | 14.1 | 229 | 2,846 | 1,046.0 | 428 | 49.8 | 22.4% |
| *Stenamma megamanni* | 754 | 858,069 | 1,138.0 | 14.5 | 217 | 3,227 | 1,166.5 | 502 | 37.8 | 28.6% |
| *Stenamma megamanni2* | 756 | 932,105 | 1,232.9 | 20.0 | 204 | 9,956 | 1,218.0 | 525 | 87.5 | 28.7% |
| *Stenamma muralla* | 734 | 830,910 | 1,132.0 | 14.8 | 221 | 3,299 | 1,113.0 | 480 | 61.6 | 30.6% |
| *Taxonus pallidicornis* | 459 | 523,674 | 1,140.9 | 21.6 | 205 | 3,001 | 1,173.0 | 282 | 37.7 | 27.5% |

**Supplemental Table 8**: Summary values describing attributes of the UCE contigs assembled by ABySS.

| **Taxon** | **UCE contigs** | **UCE contigs total BP** | **UCE contigs mean length** | **UCE contigs mean length 95 CI** | **UCE contigs min length** | **UCE contigs max length** | **UCE contigs median length** | **UCE contigs > 1kb** | **UCE contigs  coverage** | **UCE contigs  unique reads aligned** |
| --- | --- | --- | --- | --- | --- | --- | --- | --- | --- | --- |
| *Acordulecera pellucida* | 319 | 224,885 | 705.0 | 17.0 | 102 | 1,774 | 737.0 | 52 | 30.3 | 12.1% |
| *Andrena (Callandrena) asteris* | 714 | 312,069 | 437.1 | 5.5 | 102 | 901 | 431.0 | 0 | 11.5 | 36.9% |
| *Andrena (Melandrena) sp* | 705 | 449,059 | 637.0 | 9.3 | 103 | 1,577 | 635.0 | 47 | 19.9 | 17.4% |
| *Aphaenogaster albisetosa* | 302 | 197,727 | 654.7 | 19.3 | 105 | 2,072 | 621.0 | 48 | 43.9 | 2.8% |
| *Aphaenogaster megommata* | 323 | 234,220 | 725.1 | 19.5 | 103 | 1,818 | 687.0 | 63 | 46.8 | 4.3% |
| *Aphaenogaster tennesseensis* | 413 | 280,246 | 678.6 | 15.5 | 101 | 1,800 | 647.0 | 71 | 44.9 | 7.2% |
| *Aphaenogaster texana* | 348 | 181,931 | 522.8 | 13.5 | 104 | 1,714 | 496.0 | 12 | 30.3 | 5.1% |
| *Aporus niger* | 726 | 406,631 | 560.1 | 7.6 | 101 | 1,230 | 547.5 | 12 | 17.1 | 18.5% |
| *Bombus pensylvanicus* | 703 | 444,314 | 632.0 | 8.3 | 101 | 1,521 | 635.0 | 26 | 23.9 | 23.7% |
| *Chalybion californicus* | 660 | 405,336 | 614.1 | 9.5 | 101 | 1,336 | 616.0 | 34 | 34.1 | 18.1% |
| *Chyphotes mellipes* | 472 | 381,416 | 808.1 | 17.1 | 102 | 2,063 | 829.5 | 140 | 59.7 | 9.2% |
| *Evaniella semaeoda* | 515 | 361,966 | 702.8 | 12.1 | 104 | 2,191 | 721.0 | 61 | 30.9 | 22.1% |
| *Messor piceus* | 424 | 294,305 | 694.1 | 16.1 | 101 | 2,044 | 669.5 | 62 | 43.7 | 6.5% |
| *Metapolybia cingulata* | 563 | 348,507 | 619.0 | 12.4 | 101 | 1,677 | 604.0 | 62 | 38.2 | 13.2% |
| *Mischocyttarus flavitarsis* | 617 | 320,960 | 520.2 | 9.3 | 101 | 1,878 | 503.0 | 13 | 33.0 | 24.5% |
| *Nasonia vitripennis* | 756 | 350,095 | 463.1 | 8.7 | 101 | 1,368 | 450.5 | 17 | 45.5 | 22.8% |
| *Nematus tibialis* | 325 | 222,333 | 684.1 | 18.4 | 101 | 1,972 | 713.0 | 53 | 47.1 | 10.6% |
| *Orthogonalys pulchella* | 266 | 214,864 | 807.8 | 28.4 | 102 | 2,303 | 826.5 | 96 | 74.1 | 4.6% |
| *Pogonomyrmex occidentalis* | 293 | 192,855 | 658.2 | 19.9 | 106 | 2,134 | 632.0 | 36 | 56.6 | 3.1% |
| *Sapyga pumila* | 349 | 208,097 | 596.3 | 16.5 | 102 | 1,533 | 555.0 | 35 | 71.1 | 5.7% |
| *Scolia verticalis* | 516 | 409,772 | 794.1 | 13.8 | 101 | 1,857 | 803.0 | 137 | 49.2 | 16.2% |
| *Sericomyrmex harekulli* | 663 | 401,814 | 606.1 | 8.8 | 101 | 1,613 | 618.0 | 22 | 24.6 | 22.1% |
| *Stenamma diecki* | 524 | 429,972 | 820.6 | 15.2 | 101 | 1,850 | 818.0 | 164 | 46.6 | 10.0% |
| *Stenamma expolitum* | 376 | 295,389 | 785.6 | 18.5 | 101 | 2,136 | 764.0 | 95 | 50.3 | 5.4% |
| *Stenamma felixi* | 263 | 158,700 | 603.4 | 17.4 | 103 | 1,843 | 558.0 | 21 | 35.6 | 2.2% |
| *Stenamma impar* | 524 | 381,375 | 727.8 | 13.7 | 102 | 2,480 | 690.5 | 107 | 45.4 | 9.6% |
| *Stenamma megamanni2* | 314 | 240,207 | 765.0 | 20.2 | 102 | 1,980 | 719.5 | 67 | 45.6 | 3.8% |
| *Stenamma megamanni* | 545 | 437,935 | 803.6 | 15.5 | 101 | 2,653 | 793.0 | 164 | 35.1 | 12.9% |
| *Stenamma muralla* | 392 | 279,628 | 713.3 | 16.1 | 102 | 2,114 | 664.0 | 70 | 46.6 | 7.5% |
| *Taxonus pallidicornis* | 416 | 306,665 | 737.2 | 18.8 | 102 | 2,906 | 724.5 | 100 | 40.1 | 15.7% |

**Supplemental Table 9**: Model structure, AIC, number of parameters, AICc, and Akaike weight (*w*_i_) for general linear models of parameters affecting the mean number of UCE contigs captured.

|  | **model** | **AIC** | **Params** | **AICc** | **Δ_i_** | ***w*_i_** |
| --- | --- | --- | --- | --- | --- | --- |
| 1 | contigs ~ distance + reads + assembly + mean | 1241.0 | 6 | 1244.7 | 0.0 | 1.0 |
| 2 | contigs ~ distance + reads + mean | 1250.6 | 5 | 1253.1 | 8.4 | 0.0 |
| 3 | contigs ~ distance + assembly + mean | 1579.9 | 5 | 1582.4 | 337.7 | 0.0 |
| 4 | contigs ~ distance + mean | 1683.0 | 4 | 1684.6 | 439.9 | 0.0 |
| 5 | contigs ~ distance + reads + assembly | 1744.7 | 5 | 1747.2 | 502.5 | 0.0 |
| 6 | contigs ~ distance + assembly | 1755.0 | 4 | 1756.6 | 511.9 | 0.0 |
| 7 | contigs ~ reads + assembly + mean | 2593.5 | 5 | 2596.0 | 1351.3 | 0.0 |
| 8 | contigs ~ reads + assembly | 2609.5 | 4 | 2611.1 | 1366.4 | 0.0 |
| 9 | contigs ~ assembly + mean | 2629.4 | 4 | 2631.0 | 1386.3 | 0.0 |
| 10 | contigs ~ assembly | 2757.2 | 3 | 2758.1 | 1513.5 | 0.0 |
| 11 | contigs ~ distance + reads | 3147.4 | 4 | 3149.0 | 1904.3 | 0.0 |
| 12 | contigs ~ distance | 3157.7 | 3 | 3158.6 | 1914.0 | 0.0 |
| 13 | contigs ~ reads + mean | 3235.8 | 4 | 3237.4 | 1992.7 | 0.0 |
| 14 | contigs ~ mean | 3801.1 | 3 | 3802.0 | 2557.4 | 0.0 |
| 15 | contigs ~ reads | 4100.1 | 3 | 4101.0 | 2856.4 | 0.0 |

**Supplemental Table 10**: Model structure, AIC, number of parameters, AICc, and Akaike weight (*w*_i_) for general linear models of parameters affecting the number of UCE contigs captured among Trinity (only) assemblies.

|  | **model** | **AIC** | **Params** | **AICc** | **Δ_i_** | ***w*_i_** |
| --- | --- | --- | --- | --- | --- | --- |
| 1 | contigs ~ distance + mean | 424.2 | 3 | 425.2 | 0.0 | 0.8 |
| 2 | contigs ~ distance + reads + mean | 426.2 | 4 | 427.8 | 2.6 | 0.2 |
| 3 | contigs ~ distance + reads | 457.3 | 3 | 458.2 | 33.0 | 0.0 |
| 4 | contigs ~ distance | 571.9 | 2 | 572.3 | 147.2 | 0.0 |
| 5 | contigs ~ reads + mean | 829.3 | 3 | 830.3 | 405.1 | 0.0 |
| 6 | contigs ~ reads | 997.8 | 2 | 998.2 | 573.1 | 0.0 |
| 7 | contigs ~ mean | 998.8 | 2 | 999.3 | 574.1 | 0.0 |

**Supplemental Table 11**: Model structure, AIC, number of parameters, AICc, and Akaike weight (*w*_i_) for general linear models of parameters affecting the length of UCE contigs captured.

|  | **model** | **AIC** | **Params** | **AICc** | **Δi** | ***w*_i_** |
| --- | --- | --- | --- | --- | --- | --- |
| 1 | mean ~ distance + reads + assembly | 687.3 | 5 | 689.8 | 0.0 | 1.0 |
| 2 | mean ~ distance + assembly | 721.6 | 4 | 723.2 | 33.3 | 0.0 |
| 3 | mean ~ reads + assembly | 744.9 | 4 | 746.5 | 56.6 | 0.0 |
| 4 | mean ~ assembly | 775.2 | 3 | 776.1 | 86.3 | 0.0 |
| 5 | mean ~ distance + reads | 781.5 | 4 | 783.1 | 93.3 | 0.0 |
| 6 | mean ~ distance | 788.4 | 3 | 789.3 | 99.5 | 0.0 |
| 7 | mean ~ reads | 814.7 | 3 | 815.6 | 125.8 | 0.0 |

**Supplemental Table 12**: Model structure, AIC, number of parameters, AICc, and Akaike weight (*w*_i_) for general linear models of parameters affecting the length of UCE contigs captured among Trinity (only) assemblies.

|  | **model** | **AIC** | **Params** | **AICc** | **Δ_i_** | ***w*_i_** |
| --- | --- | --- | --- | --- | --- | --- |
| 1 | contig length ~ distance + reads | 333.1 | 3 | 334.0 | 0.0 | 1.00 |
| 2 | contig length ~ distance | 373.6 | 2 | 374.0 | 40.0 | 0.00 |
| 3 | contig length ~ reads | 375.8 | 2 | 376.3 | 42.3 | 0.00 |

**Supplemental Figure 1**: Maximum likelihood phylogeny inferred from a 75% complete supermatrix containing data from ultraconserved elements identified in 14 genome-enabled taxa. We show bootstrap support values only where support is < 100%. Although genome assemblies exist for additional hymenopteran taxa, we were not granted permission to include these data in our analyses.

**Supplemental Figure 2**: Box plots showing differences in standard metrics among UCE contigs assembled by Trinity or ABySS. Jittered dots indicate a given value for each taxon. Values correspond to those in Supplemental Table 7 and Supplemental Table 8.

**Supplemental Figure 3**: Maximum likelihood phylogeny inferred from a 75% complete supermatrix containing data from 14 genome-enabled taxa (identified by double-asterisks) and 30 taxa from which we enriched and assembled (ABySS) ultraconserved element loci. We show bootstrap support values only where support is < 100%, and the single asterisk beside *Stenamma megamanni* denotes that this sample represents a different population of the same species.

**Supplemental Figure 4**: The topology from Figure 1, with branches colored to indicate the approximate number of ultraconserved element loci we captured, by taxon, relative to the total number of loci captured from *Nasonia vitripennis* (n=1,166).

**Supplemental Figure 5**: Bar plots comparing parameter (β) estimates (± 95% CI) from general linear models of factors affecting the number of UCE contigs enriched and the length of enriched UCE contigs. Note that the y-axis differs across sub-panels.

**Supplemental Figure 6**: Maximum likelihood phylogeny inferred from a 75% complete supermatrix containing data from 14 genome-enabled taxa (identified by double-asterisks) and 27 taxa from which we enriched and assembled (Trinity) ultraconserved element loci. To infer this tree, we removed three sawfly taxa from consideration, re-identified UCE loci, re-extracted relevant UCE contigs, and re-aligned the extracted data, resulting in a slightly larger data matrix from that in Figure 1. We show bootstrap support values only where support is < 100%, and the single asterisk beside *Stenamma megamanni* denotes that this sample represents a different population of the same species.
